# A dual efficacy-implementation trial of a novel mobile application for childhood nephrotic syndrome management: the UrApp for childhood nephrotic syndrome management pilot study protocol (UrApp pilot study)

**DOI:** 10.1186/s12882-020-01778-w

**Published:** 2020-04-09

**Authors:** Chia-shi Wang, Cam Escoffery, Rachel E. Patzer, Courtney McCracken, Diana Ross, Michelle N. Rheault, Amira Al-Uzri, Larry A. Greenbaum

**Affiliations:** 1 Department of Pediatrics, Emory University School of Medicine, Atlanta, Georgia USA; 2 Children’s Healthcare of Atlanta, Atlanta, GA USA; 3 Department of Behavioral Sciences and Health Education, Emory University Rollins School of Public Health, Atlanta, GA USA; 4 Department of Surgery, Emory University School of Medicine, Atlanta, GA USA; 5 University of Minnesota Masonic Children’s Hospital, Minneapolis, GA USA; 6 Oregon Health and Science University, Portland, OR USA

**Keywords:** Nephrotic syndrome, Mobile application, Self-management, Adherence, Child

## Abstract

**Background:**

Idiopathic nephrotic syndrome has a relapsing-remitting course in the majority of pediatric patients, demanding vigilant monitoring and self-management. A novel, expert-designed, user-informed mobile application (app), UrApp©, was created to support management tasks, including home urine protein monitoring.

**Methods:**

The UrApp Pilot Study (ClinicalTrials.gov, NCT04075656) is a randomized trial comparing UrApp-supported nephrotic syndrome management with standard-of-care with parallel process evaluation of the intervention delivery. Sixty caregivers of children with newly diagnosed, steroid-sensitive nephrotic syndrome will be randomized 1:1 to UrApp-supported care or standard-of-care. Follow-up will be 1 year, with primary outcomes of adherence to urine monitoring and medications assessed at 6 and 12 months. Secondary outcomes at 6 and 12 months include self-efficacy, quality-of-life, hospitalizations and delayed relapse diagnoses. A mixed-methods approach will evaluate UrApp engagement, use retention, features used, user perceptions, and contextual barriers and facilitators of UrApp use. User behavior will be assessed for relationships to the primary and secondary outcomes. A Stakeholder Committee of volunteer trial participants, clinicians, and engineers will examine the trial results and design a pragmatic UrApp-enhanced nephrotic syndrome intervention with potential for wide implementation. The final UrApp intervention will be tested in a user-centered hybrid effectiveness-implementation trial designed with stakeholder input.

**Discussion:**

The UrApp Pilot Study examines the efficacy of a novel app designed specifically for nephrotic syndrome. The protocol involves dual efficacy and process evaluation aims to increase efficiency and incorporates the stakeholders’ perspective in formative assessment to inform intervention redesign and the design of a future user-centered trial.

**Trial registration:**

ClinicalTrials.gov, NCT04075656. Registered on September 2, 2019, https://clinicaltrials.gov/ct2/show/NCT04075656

## Background

Idiopathic nephrotic syndrome (NS) is one of the most common chronic kidney diseases in children, with a prevalence of approximately 16 cases per 100,000 children [1]. Though > 80% of children will achieve remission with corticosteroid treatment, the majority will experience subsequent relapses, with more than half relapsing frequently or becoming dependent on corticosteroids to maintain remission [2–5]. During a relapse, patients can develop complications such as anasarca, acute kidney injury, serious infections, or thromboembolic events, necessitating hospitalization [6]. In the U.S. in 2006 and 2009, childhood NS resulted in an estimated 48,700 inpatient days and charges totalling $259 million [7].

Management of children with NS entails long-term outpatient surveillance and treatment. Home care includes the important standard-of-care task of urine protein monitoring to follow the relapsing-remitting nature of the disease using urine test strips. New proteinuria signals disease relapse *before* the development of overt symptoms such as edema. When alerted of a new relapse, providers can prescribe corticosteroids or other medications to treat the relapse and prevent acute disease complications and hospitalizations. Patients and their family members can also track urine protein for resolution so that treatments can be stopped or reduced to minimize toxicity [8]. For patients with significant corticosteroid side effects (e.g., hypertension, growth retardation, obesity), second-line agents are prescribed, which have additional side effects that require ongoing monitoring. Optimal NS disease management thus demands a high level of active patient/caregiver participation and vigilance to monitor the course of disease and communicate with providers. Not unlike other chronic, relapsing-remitting pediatric disorders, self-management is difficult for NS patients and their caregivers. Adherence to urine protein testing and medications are low [6, 9]. In a focus group and individual interview study, parents reported difficulty understanding the clinical meaning of urine protein results and knowing when to report and act on urine protein changes [10]. These issues in self-management may have a direct impact on disease outcome, in that self-reported medication nonadherence during initial induction treatment was associated with delayed disease remission [9]. However, there are no evidence-based tools to support NS management.

**Fig. 1:**
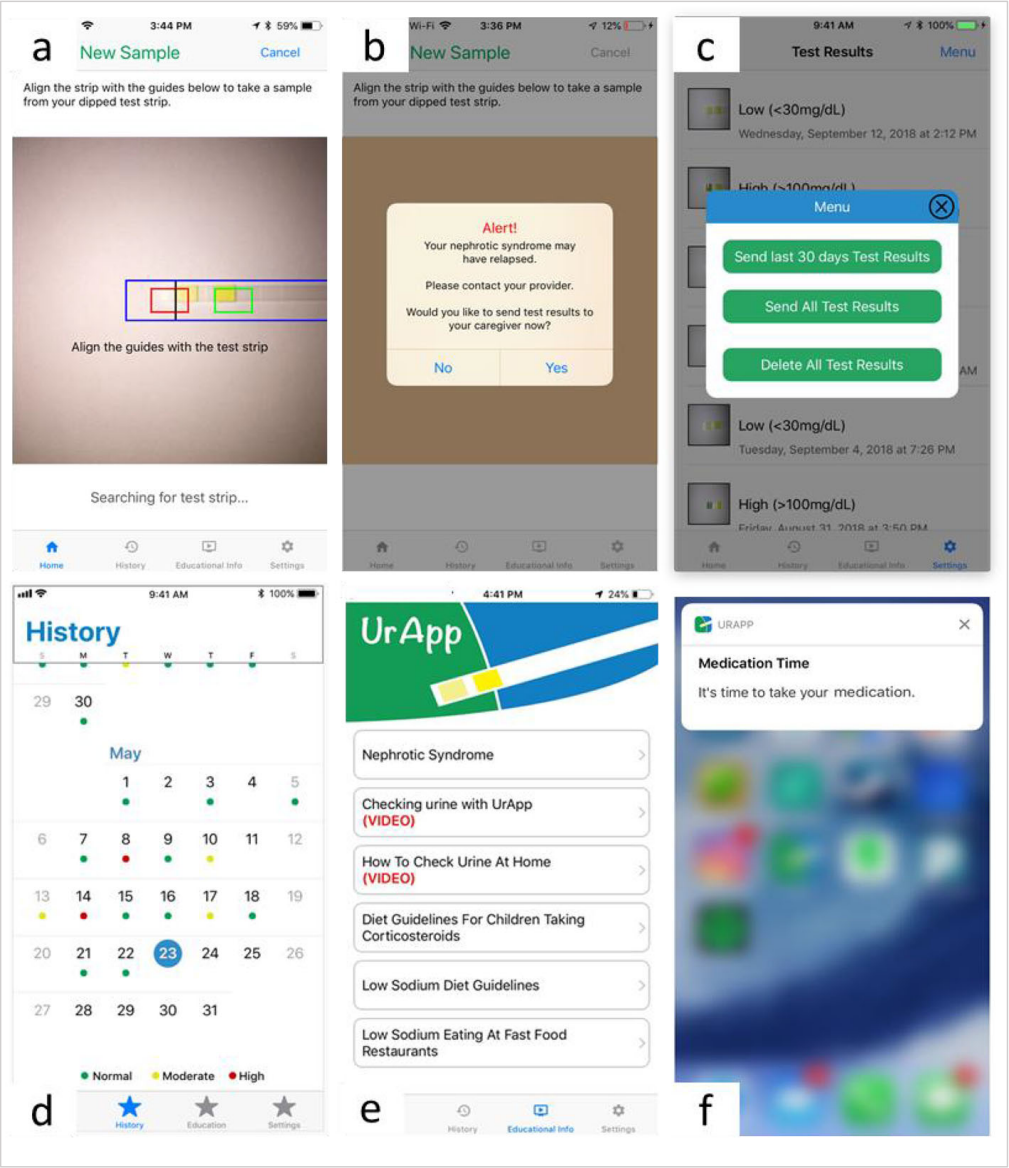
UrApp screenshots displaying key functions: **a** camera read of urine test strips, **b** alerts for significant urine protein findings, **c** transmission of urine protein results, (d) urine protein documentation, **e** educational materials, and **f** medication reminders [11]

Recently, our team developed a novel mobile application (app) to support patients and their caregivers with NS disease management, UrApp©. UrApp© is freely available for download at: https://apps.apple.com/us/app/urapp-nephrotic-syndrome-mgr/id1446483252. The app was iteratively developed by an expert panel of pediatric nephrologists and mobile health app engineers and refined by two rounds of usability testing and user feedback [11]. Features include: camera read of urine test strips, analysis and display of urine protein trends, alerts for new disease relapse or newly achieved disease remission, transmission of urine protein results to providers, patient education materials, and reminders for medications and urine testing (Fig. 1). We hypothesize that the use of an app that reads urine dipsticks, interprets trends in results, and communicates the results to providers will facilitate improved communication about relapse status between patients and caregivers and providers, hopefully decreasing disease complications and minimizing corticosteroid exposure. Here, we describe our protocol to assess the preliminary efficacy and feasibility of implementation of UrApp in The UrApp for Childhood Nephrotic Syndrome Management Pilot Study (ClinicalTrials.gov Identifier: NCT04075656).

## Methods/design

### Overall design

The UrApp Study is a pilot randomized clinical trial comparing UrApp-supported management (UrApp Arm) to standard-of-care (SOC Arm) with concurrent process evaluation to gain understanding into UrApp uptake, delivery, and influences on its efficacy. Table 1 displays the outcome measures relevant to clinical efficacy and feasibility of implementation. A Stakeholder Committee will be created to review study results in a formative assessment in order to refine the UrApp intervention and plan a future pragmatic, user-centered, hybrid effectiveness-implementation trial. This committee will be composed of trial participants, clinical study investigators (CW, MNR, AA), and engineers.

**Table 1:** Clinical efficacy and feasibility outcome measures in the UrApp for Childhood Nephrotic Syndrome Management Pilot Study

Efficacy	Implementation
Medication adherence	UrApp frequency of use
Adherence with urine testing	UrApp use over time
Caregiver self-efficacy	UrApp functions used and frequency
Patient qualify-of-life	User perceptions of UrApp
Caregiver qualify-of-life	Barriers of UrApp use
Hospitalizations	Facilitators of UrApp use
Delayed relapse detection	

### Pilot trial design

#### Setting

Participants will be recruited from the nephrology clinics of three large pediatric nephrology centers in the United States: Children’s Healthcare of Atlanta (CHOA), University of Minnesota (UMn), and Oregon Health and Science University (OHSU).

#### Participants

Sixty caregivers of patients ages 1–17 with newly diagnosed steroid sensitive NS will be enrolled. Steroid sensitive NS is defined clinically by edema; nephrotic range proteinuria (urine protein to creatinine ratio > 2 mg/mg, or ≥ 300 mg/dL or ≥ 3+ protein on urine dipstick); hypoalbuminemia ≤2.5 g/dL; and resolution of proteinuria within 4 weeks of corticosteroid treatment. Additional inclusion criteria are: NS diagnosis made within 42 days at time of enrolment; ownership of at least one functioning Apple iPhone with internet/WIFI access in the family; and caregiver proficiency with English. Exclusion criteria are: end-stage kidney disease, renal transplantation, and clinical or histologic evidence of secondary NS (e.g., systemic lupus erythematosus).

Sample size of 60 participants (randomized 1:1) will achieve 82% power to detect a 35% point difference in the proportion of patients adherent with urine monitoring (primary outcome), with 20% drop-out. Sample size and power estimates are based on 55% adherence in the control group and 90% in the UrApp intervention group. These estimates are based on our single center experience with SOC [6] and our experience with text messaging for NS monitoring, where 94% of the participants continued to check urine and provide results via text messaging at the end of 1 year [12]. Power was calculated using a two-sided Z-test with pooled variance at 0.05 type I error rate using PASS v. 14.0.8 (Kaysville. UT).

#### Procedures

Participants will be enrolled and randomized 1:1 to UrApp or SOC arms at the baseline visit between 2 and 6 weeks after diagnosis. Participant follow-up will be 1 year. Caregiver consent and patient assent will be obtained per local institutional review board (IRB) guidelines by site coordinators in person.

Randomization will be created by a biostatistician blinded to treatment allocation. She will generate a random allocation sequence using a pseudo-random-number generator with randomly permutated blocks stratified by study center. Assignments will be placed in sealed, sequenced, opaque envelopes, which study coordinators will open at time of enrolment to assign participants to SOC or UrApp.

SOC Arm participants will be provided a folder of educational material on NS, including general information on symptoms, treatments, and possible complications; healthy diet for children taking corticosteroids; and low sodium diet. Site research staff will demonstrate how to check urine for protein with test strips, and educate patients on the definitions of disease relapse and remission [8]. Urine test strips and urine protein logs will be provided to ensure that each participant can check their urine daily for protein. Participants will be instructed to check their urine daily for protein and contact their provider within 1 business day for relapses and remissions. Since the majority of patients with NS are between 2 and 7 years of age, [13] participant education and materials provided will be targeted towards patient caregivers. Once in months 1–3 and 5–7, site research staff will contact the caregivers by telephone or in-person during a routinely scheduled clinic appointment to follow up on any questions regarding NS in general or urine monitoring. Any disease specific or treatment specific questions will be directed to the treating physician. Surveys will be administered by research staff in person at baseline, 6 months (+/− 1 month), and 12 months (+/− 1 month). We will attempt to administer the surveys at routinely scheduled clinic appointments, with the option of administering the surveys online if appointments do not fall within the timeframes.

UrApp Arm participants will follow the same procedures as described above for the SOC arm. In addition, caregivers/parents will download UrApp at the baseline visit. UrApp contains instructional videos to guide users. Participants will be instructed to test use the app, and questions/difficulties using the app will be recorded by site research staff and resolved. The telephone number of the provider’s office for the patient will be entered into UrApp. Participants will be able to call their provider’s office directly through UrApp. The email addresses of the study staff will also be entered. UrApp will automatically e-mail test results to the research staff when elected by the users. During the planned contacts in months 1–3 and 5–7, as described for the SOC arm, research staff will inquire about any questions/technical issues with UrApp. These issues will be recorded and resolved. Participants will be asked by study staff, and reminded by the app, to call their providers and send urine testing results to the study staff whenever there is a relapse or remission. When the study staff receives alerts of a relapse/remission via UrApp, the information will be communicated to the treating physician within 1 business day.

Participants will receive $20 for each completed study visit.

#### Measures

**Fig. 2:**
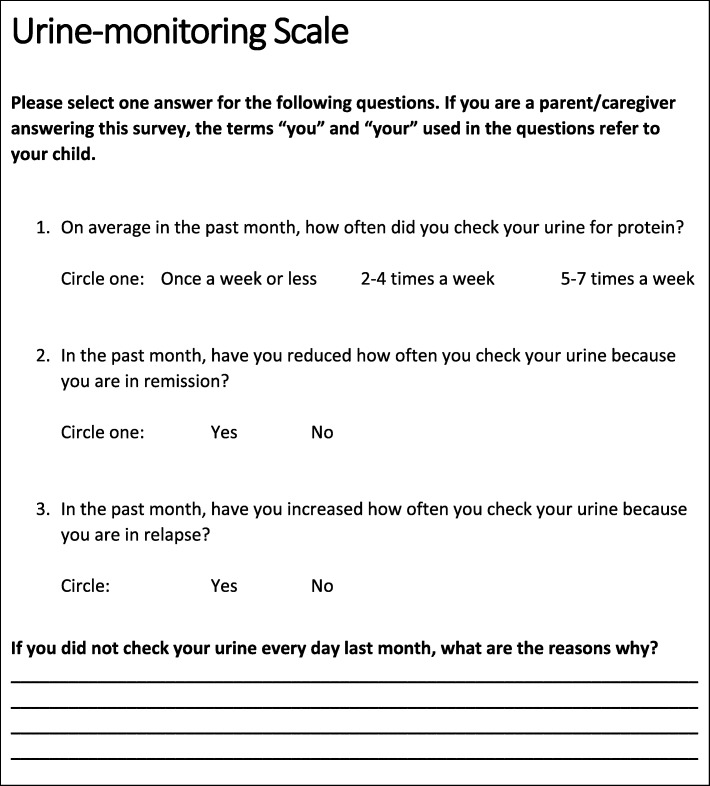
Urine-monitoring survey for nephrotic syndrome

Baseline measures will be collected at the baseline visit between 2 and 6 weeks after NS diagnosis. *Demographic characteristics* of the patient caregiver (UrApp user) and the patient will be collected from the medical chart and by participant questionnaire. Variables will include: caregiver age, gender, race/ethnicity, income level, and educational level; patient age, gender, and race/ethnicity. As one inclusion criteria for trial participation is ownership of an iPhone, examination of demographic variables will provide information on the representativeness of the cohort to the general NS population and the generalizability of study findings. Baseline *medication adherence*, and *testing adherence, self-efficacy*, and *quality of life (QoL)* will be assessed by surveys. Adherence to medications will be evaluated via caregiver survey with the validated four question Morisky, Green, and Levine (MGL) Adherence Scale [14]. Adherence with urine monitoring will be evaluated with a newly created questionnaire with face validity assessed by two pediatric nephrologists (CW and LG) and one behavioral science expert (CM), as no instrument specific to urine testing is currently available (Fig. 2). Caregiver self-efficacy will be assessed by a survey adapted from the Self-Efficacy for Managing Chronic Disease 6-Item Scale (Stanford Patient Education Research Center). The survey will include 3 questions with a scale of 1 = not at all confident to 10 = totally confident. Patient/caregiver-reported QoL will be assessed by using the 23-item Patient-Reported Outcomes Measurement Information System - Pediatric Quality of Life Inventory (PedsQL). PedsQL was developed as part of the NIH Roadmap Initiative to create universal measures for patient-reported outcomes, and contains questions in the domains of social-peer, depression, anxiety, mobility, and function. The survey has been found to have validity and feasibility in children and adolescents with NS [15]. Caregivers and patients ages 5–17 will be surveyed with parent or patient forms, respectively.

Outcome measures will be collected at 6 and 12 months via caregiver surveys, medical chart review, and urine protein logs (UrApp data logs for the UrApp arm). Medication and urine testing adherence, self-efficacy, and QoL surveys will be administered in the same way as during the baseline visit. In addition, UrApp data log (UrApp arm) and urine protein logs (SOC arm) will also be collected to obtain the frequency of monitoring. A priori, we define adherence with urine protein monitoring as checking, on average, at least 2 times a week in the month preceding the assessment. *Relapse detection*: patient medical charts will be reviewed for occurrence and frequency of delayed relapse reporting, which is defined as a relapse not reported to the treating physician until clinical manifestations or complications have occurred and/or only discovered during planned or unplanned visits or hospitalizations. *Hospitalizations*: medical records will be reviewed for the primary reason for admission and NS disease complications diagnosed during the hospitalization, including bacterial peritonitis, septicemia, shock, blood clot(s), acute kidney injury, and seizures from hyponatremia or hypertension.

ANALYSIS will follow the intention-to-treat principle to preserve the integrity of the randomization. Primary outcomes, adherence with urine monitoring and medications, will be analyzed via Chi-square test to compare the proportions of adherent patients in the UrApp vs SOC arms at 6 and 12 months. Results will be presented as group proportions and difference in proportions with associated 95% CIs. We will explore changes in adherence over time in an adjusted analysis using a generalized linear mixed model, by testing the treatment arm (UrApp vs. SOC) by time (6 and 12 months) interaction. Models will include site as a random effect and adjust for any baseline characteristic differences between groups (including sex as a variable). Self-efficacy and QoL measures will be analyzed using linear mixed models. Models will include the effect of treatment (UrApp vs. SOC), time (baseline, 6 and 12 months), and the treatment by time interaction. Study center will be included as a covariate and/or a random effect. Post-hoc pairwise comparisons will be used to compare specific points in follow-up (6 and 12 months) to baseline within and across groups. Results will be presented as differences in means with associated 95% CIs. Baseline characteristics will be included in the models as covariates in an adjusted analysis. Three-way interactions will be utilized to explore the role of demographic characteristics and their association with treatment response. While not adequately powered to conduct a moderator analysis, effect sizes will be calculated by subgroup and will inform a future, full-scale study. Delayed relapse diagnosis and hospitalizations will be compared between the treatment arms using Poisson regression under the framework of generalized linear mixed models or generalized estimating equations to account for correlated data collected from the same individuals. Baseline characteristics will be included in the models as covariates. Analysis will carried out using SAS v.9.4 (Cary, N.C.). Statistical significance will be assessed at the 0.05 level.

### Process evaluation design

Process evaluation examines the extent to which an intervention is implemented as intended and the influences on its effectiveness, and is increasingly recognized as integral to both the development and assessment of health promotional programs [16]. Our UrApp-based intervention is novel in the childhood NS field; hence, we aim to conduct a process evaluation alongside the pilot randomized trial to increase the rigor of this formative process to design an effective management support tool. One of the main purposes of a process evaluation is to determine the extent to which an intervention was delivered as intended. Key elements include: *fidelity* (delivery as planned) and *dose* (quantity of the intervention delivered and extent to which participants actively engaged with the materials including initial and continued use) [16, 17]. We operationalize these concepts as: UrApp engagement (frequency of use [*dose*]), retention (use over time [*dose*]), and app functions used and frequency of use (depth of use [*fidelity and dose*]). A second main purpose of process evaluation is to understand the contextual influences (i.e. barriers and facilitators) and user perceptions on an intervention’s future implementation and success.

#### Procedures

User behavior data will be captured by software analytics and include information on: *frequency of use*, *frequency of use over time*, and *specific app functions used and frequency*. These measures will be downloaded at 6 and 12 months among participants in the UrApp arm (*n* = 30).

User perceptions of UrApp features and contextual barriers and facilitators of UrApp use will be collected via surveys and interviews of the 30 participants randomized to the UrApp arm at 6 and 12 months of trial participation. Interviews will be conducted either in person or over the telephone with audio recording. Interviews will follow a semi-structured guide and will last approximately 30 min. Questions will target perceptions of the usefulness, satisfaction, and ease of use of UrApp and its specific features. Barriers and facilitators of UrApp use will be queried, as well as recommendations for changes to UrApp. The interviewers will lead with open-ended questions, with follow-up questions probing for understanding. Participants will receive $50 for completed interviews.

#### Measures

Participant surveys on the *general perceptions of mobile health apps* will be obtained at baseline. A survey on the *perceived usefulness, satisfaction, and ease of use of UrApp* will be administered to caregivers at 6 and 12 months in the UrApp arm. Surveys items will be adapted from the USE Questionnaire, a survey designed specifically to measure user perceptions of mobile apps and other user support products [18].

#### Analysis

A priori, we define adequate engagement as accessing UrApp at least two times per week during the first 3 months, poor retention as a 50% decrease in weekly use over time, and adequate depth of use as transmission of data to providers for all episodes of disease relapse/remission when prompted. The relationship between user behavior and primary and secondary outcomes (detailed under Aim 1) will be assessed via Chi-square test/Fisher’s exact test or t-tests or Wilcoxon rank-sum tests, as appropriate.

Survey results will be tabulated and described for each study time-point. Baseline perceptions of mHealth apps and caregiver demographic characteristics will be examined for associations with UrApp perceptions at 6 and 12 months using Chi-square tests and two-sample t-tests or Wilcoxon rank-sum tests, as appropriate.

Analysis of interviews will involve multiple steps to ensure data validity and reliability [19]. The interviews will be transcribed verbatim. Transcripts will be analyzed using thematic analysis by identifying deductive and inductive themes. Transcripts will be coded by 2–3 independent coders using a qualitative data analysis software program (i.e., NVivo). A codebook will be developed for concise definition and to ensure consistency in code application. A pediatric nephrologist and the lead investigator (CW) will identify an initial list of deductive themes. Inductive, data driven themes and concepts will be added as they emerge from the transcripts. Inter-coder agreement (2 coders) will be assessed prior to coding the whole dataset and discrepancies rectified. Data collection will stop once data saturation is reached (i.e., no new codes). Categories and sub-categories of themes from the qualitative interviews will be derived from the codes.

### Stakeholder committee

Stakeholder engagement is an effective framework for ensuring that research is patient-centered, with results that have greater relevance and impact for the intended end users [20]. In this formative process of developing the first-of-its-kind mobile health tool for NS management, we will integrate stakeholder involvement in the evaluation of the pilot trial results and plans for UrApp refinement and a future full-scale clinical trial. Our procedural approach incorporates elements of the Patient-Centered Outcomes Research Institute Engagement Rubric: *reciprocal relationships* (including partners as key personnel)*, partnership* (fair compensation, reasonable and thoughtful requests for time, inclusiveness and respect)*, co-learning* (researchers help patients and other stakeholders to understand the research process, patient-centeredness and stakeholder engagement incorporated)*, transparency-honesty-trust* (inclusive decision making, information is readily shared, commitment to open and honest communication) [21]. This will provide key multidisciplinary perspectives, including the voice of the patient caregiver, to increase the likelihood of a patient-responsive care management intervention and meaningful clinical trial.

#### Procedures

Stakeholder members will include four pediatric nephrologists, three research engineers with human-computer interaction expertise who developed UrApp and are intimately aware of technical possibilities/restrictions, and five patient caregivers in the UrApp arm who have completed 12 months of follow-up. Participants in the UrApp arm at the lead site (CHOA) will be approached for interest in joining the stakeholder committee during the pilot trial. Four face-to-face stakeholder meetings with a telephone conference option for members who cannot be present will be conducted. Meetings will be held every 4–6 months, with the first meeting starting at the end of pilot trial recruitment, when we anticipate having 6-months follow-up data on at least half of the trial participants.

Caregiver members will be compensated $75 for each meeting, and food/drinks will be provided (fair compensation under *partnership*). Meeting 1 will focus on introducing members to each other and building relationships (*reciprocal relationships* and *trust*). We will outline the goals of the stakeholder committee to refine UrApp and plan a future trial (*co-learning*). A summary of the trial results to date on UrApp efficacy and process evaluation will be shared (*transparency*, *honesty*), and members will be invited to share their perspectives and interpretation of the trial results (*co-learning*). Meetings 2–4 will include a review of updated trial results. Informed by the trial results, the committee will iteratively plan UrApp refinement (*transparency, co-learning,* and *reciprocal relationships*). Caregivers will provide input on outcomes most important to users. The investigators will use the feedback to iteratively develop measures/procedures to be reviewed at subsequent meetings (*co-learning, reciprocal relationships*). Caregivers will provide feedback on the protocols to improve recruitment and retention.

#### Data management

REDCap, a secure data collection system that encrypts all data, will be used for data collection and management for all data collected from surveys and the medical charts. Interview transcripts will be stored digitally in a password-protected computer. Interview analysis will use software, NVivo, which provides data encryption. Access to both systems will be controlled by a sequence of individually assigned user identification codes and passwords, made available only to authorized personnel who have completed prerequisite training. Site staff will receive training prior to receiving access to REDCap for data entry. The lead site investigator and study administer will query data monthly and review entries for completeness and accuracy.

#### Safety monitoring

Screening for adverse events will be performed at each study contact, either by phone or in person. Targeted questions will screen for issues with using the mobile app as well as common nephrotic syndrome disease complications (e.g. edema).

An external Data Monitoring Committee (DMC) has been created prior to the randomization of the first patient. The DMC will perform safety reviews every 6 months unless otherwise requested by the Chair of the DMC. The DMC will also receive reports on a regular basis on all serious adverse events (SAEs) reported for this trial. One interim analysis and one final analysis are planned. The study would be stopped if the DMC and the lead PI believe that there is a significant safety issue or that the interim analysis indicates that completing the study is not appropriate.

A SAE is defined as: death; life-threatening event; requires or prolongs hospitalization; results in disability significant, persistent, or permanent; pregnancy with a resultant birth defect; causes cancer; or overdoses of a study medication. In addition to the completion of the appropriate forms, any serious adverse event occurring during the study will be reported to the primary investigator (PI, CW), the DMC, the local IRB, and the participating sites. The PI will make an assessment of whether the event constitutes an unanticipated problem posing risks to subjects or others. This assessment will be provided to the lead site IRB, which, in turn will make a final determination and notify the appropriate regulatory agencies and institutional officials.

Adverse events that do not meet criteria for SAE will be reported using the Adverse Event Form during the follow-up visit. Information regarding the diagnosis, date of occurrence, severity of the event, and therapy will be recorded. A determination of the relationship of the adverse event to study participation will be made by the site PI. Should a relationship be found, results will be reported to the lead PI DMC within 1 business week.

## Discussion

The present protocol represents one of the first formative efforts to rigorously test and refine a technically innovative mobile health app designed specifically for nephrotic syndrome management. UrApp® was designed by an expert panel with input from patients and caregivers and demonstrates excellent analytic validity and usability [11]. Our goals are to assess the efficacy of UrApp®, gather information on the implementation of this novel intervention, and continue to involve the critical stakeholder perspectives into intervention redesign and future testing. The successful completion of this study will produce one of the first evidence-based tools for the childhood nephrotic syndrome population. The trial is currently enrolling.

## Data Availability

All study documents including consent and assent forms will be available from the corresponding author upon reasonable request. Trial registration on ClinicalTrials.gov (NCT04075656) was made on September 2, 2019. All study results will be submitted to ClinicalTrials.gov as per NIH policy.

## Abbreviations

App: Application
CHOA: Children’s Healthcare of Atlanta
DMC: Data Monitoring Committee
IRB: Institutional review board
MGL: Morisky, Green, and Levine (MGL) Adherence Scale
NS: Nephrotic syndrome
OHSU: Oregon Health and Science University
PedsQL: Patient-Reported Outcomes Measurement Information System - Pediatric Quality of Life Inventory survey
PI: Primary investigator
QoL: Quality of life
SAE: Serious adverse event
SOC: Standard of Care arm
UMn: University of Minnesota

## References

[1] Eddy AA, Symons JM (2003). Nephrotic syndrome in childhood. Lancet.

[2] Primary nephrotic syndrome in children: clinical significance of histopathologic variants of minimal change and of diffuse mesangial hypercellularity. A Report of the International Study of Kidney Disease in Children. Kidney Int. 1981;20(6):765–71. 10.1038/ki.1981.209 7334749

[3] The primary nephrotic syndrome in children. Identification of patients with minimal change nephrotic syndrome from initial response to prednisone. A report of the International Study of Kidney Disease in Children. J Pediatr. 1981;98(4):561–4. 10.1016/s0022-3476(81)80760-3 7205481

[4] Greenbaum LA, Benndorf R, Smoyer WE (2012). Childhood nephrotic syndrome--current and future therapies. Nat Rev Nephrol.

[5] Lombel RM, Gipson DS, Hodson EM (2013). Kidney disease: improving global O: treatment of steroid-sensitive nephrotic syndrome: new guidelines from KDIGO. Pediatr Nephrol.

[6] Wang CS, Yan J, Palmer R, Bost J, Wolf MF, Greenbaum LA (2017). Childhood Nephrotic syndrome management and outcome: a single center retrospective analysis. Int J Nephrol.

[7] Gipson DS, Messer KL, Tran CL, Herreshoff EG, Samuel JP, Massengill SF, Song P, Selewski DT (2013). Inpatient health care utilization in the United States among children, adolescents, and young adults with nephrotic syndrome. Am J Kidney Dis.

[8] Lombel RM, Gipson DS, Hodson EM (2013). Kidney disease: improving global outcomes: **treatment of steroid-sensitive nephrotic syndrome: new guidelines from KDIGO**. Pediatr Nephrol.

[9] Wang CTJ, Srivastava T, Weidemann D, Greenbaum LA. Medication adherence and perceived difficulties in pediatric nephrotic syndrome. In: *ASN*. 2017;2017.

[10] Beanlands H, Maione M, Poulton C, Herreshoff E, Hladunewich MA, Hailperin M, Modes MM, An L, Nunes JW, Trachtman H (2017). Learning to live with nephrotic syndrome: experiences of adult patients and parents of children with nephrotic syndrome. Nephrol Dial Transplant.

[11] Wang CS, Boyd R, Mitchell R, Wright WD, McCracken C, Escoffery C, Patzer RE, Greenbaum LA (2019). Development of a novel mobile application to detect urine protein for nephrotic syndrome disease monitoring. BMC Med Inform Decis Mak.

[12] Wang C-s, Troost JP, Greenbaum LA, Srivastava T, Reidy K, Gibson K, Trachtman H, Piette JD, Sethna CB, Meyers K (2019). Text messaging for disease monitoring in childhood Nephrotic syndrome. Kidney International Reports.

[13] Nephrotic syndrome in children: prediction of histopathology from clinical and laboratory characteristics at time of diagnosis. A report of the International Study of Kidney Disease in Children. Kidney Int. 1978;13(2):159–65. 10.1038/ki.1978.23 713276

[14] Morisky DE, Green LW, Levine DM (1986). Concurrent and predictive validity of a self-reported measure of medication adherence. Med Care.

[15] Gipson DS, Selewski DT, Massengill SF, Wickman L, Messer KL, Herreshoff E, Bowers C, Ferris ME, Mahan JD, Greenbaum LA (2013). Gaining the PROMIS perspective from children with nephrotic syndrome: a Midwest pediatric nephrology consortium study. Health Qual Life Outcomes.

[16] Moore GF, Audrey S, Barker M, Bond L, Bonell C, Hardeman W, Moore L, O'Cathain A, Tinati T, Wight D (2015). Process evaluation of complex interventions: Medical Research Council guidance. BMJ.

[17] Saunders RP, Evans MH, Joshi P (2005). Developing a process-evaluation plan for assessing health promotion program implementation: a how-to guide. Health Promot Pract.

[18] Lund A (2001). Measuring usability with the USE questionnaire. Usability Interface.

[19] Hennink M, Hutter I, Bailey A (2011). Qualitative research methods.

[20] Forsythe L, Heckert A, Margolis MK, Schrandt S, Frank L (2018). Methods and impact of engagement in research, from theory to practice and back again: early findings from the Patient-Centered Outcomes Research Institute. Qual Life Res.

[21] Sheridan S, Schrandt S, Forsythe L, Hilliard TS, Paez KA (2017). Advisory panel on patient E: the PCORI engagement rubric: promising practices for partnering in research. Ann Fam Med.

